# Psychometric properties of the WHOQOL-BREF(PT) in a sample of elderly citizens

**DOI:** 10.1186/s12955-021-01783-z

**Published:** 2021-05-17

**Authors:** Margarida Goes, Manuel Lopes, João Marôco, Henrique Oliveira, César Fonseca

**Affiliations:** 1 School of Health, Polytechnic Institute of Beja, Beja, Portugal; 2 Comprehensive Health Research Centre (CHRC), Universidade de Évora, Évora, Portugal; 3 ISPA – Instituto Universitário de Ciências Psicológicas, Sociais e da Vida, Lisbon, Portugal; 4 Instituto de Telecomunicações (IT), Lisbon, Portugal

**Keywords:** Elderly, Quality of life, WHOQOL-BREF, Psychometric properties, Structural equation modelling

## Abstract

**Background:**

The goal of this article was to research the psychometric properties of the WHOQOL-BREF(PT) instrument in a sample of elderly citizens residing in a rural area in their own homes or at family members’ or friends’ homes and to compare the results: (i) to those reported by the team of Portuguese researchers that undertaken the instrument's translation/validation to the Portuguese language and (ii) to those reported internationally by the World Health Organization Quality of Life group. An overall quality of life scoring (QOL_24_—all facets) is also proposed in this article as novelty. The correlation level between QOL_24_ and the instrument’s general facet was also investigated.

**Methods:**

This was a cross-sectional study with a sample of 351 elderly citizens (46.4% males and 53.6% females) randomly selected from the official dataset of the Local Health Unit of Baixo Alentejo. All the data were collected by health professionals at the participants’ homes following the structured interview methodology and using the WHOQOL-BREF(PT) instrument. Three different structural equation models were developed: (i) a first-order confirmatory factor analysis, to assess the instrument’s psychometric properties; (ii) a hierarchical second-order confirmatory factor analysis model, to allow determining the QOL_24_ scoring; and (iii) a more generic structural equation model, to investigate the correlation level between QOL_24_ and the instrument’s general facet.

**Results:**

The WHOQOL-BREF(PT) showed an “almost very good” goodness of fit (comparative fit index of 0.949 and Tucker-Lewis index of 0.943), an adequate internal consistency (Cronbach’s alpha: from 0.64 to 0.90; composite reliability: from 0.59 to 0.88) and tolerable convergent validity (average variance extracted: from 0.374 to 0.614). However, discriminant validity was not reached because strong correlations between the first-order factors (four QOL domains) were obtained, together with low values of the average variance extracted. The scoring of QOL domains and QOL_24_, determined as weighted averages (proposed in this article as novelty) were significantly different than those determined as unweighted averages. The standardized correlation coefficient between QOL_24_ and the instrument’s general facet was of 0.89 (statistically highly significant).

**Conclusions:**

The WHOQOL-BREF(PT) is a psychometrically sound instrument to assess the QOL of the considered population sample. However, the QOL domains were found strongly intertwined. More studies are necessary to validate the weighted average scoring strategy of QOL domains and QOL_24_. Concurrent validity between QOL_24_ and the instrument’s general facet was considered as “strong”.

## Background

Organizations and institutions worldwide, as well as the latest scientific research, have shown an increase in individuals’ average life expectancy, which is one of the features that has been used to explain the phenomenon of population aging [1, 2]. However, the additional years of life gained come with a higher incidence/prevalence of noncommunicable diseases (multiple and complex comorbidities) and disabilities [3, 4], reflected in the increase in populations’ disease burden [5], mainly in older age groups [6, 7]. Consequently, a greater demand in managing age-related health conditions has been widely emphasized, mainly through the implementation of skilled healthcare services [8].

Even so, the major goal in the delivery of effective health care to patients with comorbidities and disabilities consists of reducing the impact of their illnesses, not only in terms of physical functioning but also on the different dimensions of their life [9]. But the uncertainty about disease diagnosis and prognosis, disease progression and the treatments that need to be delivered are aspects that, inevitably, may cause some degree of emotional disturbance that can affect individuals' life. Such emotional disturbance may result in significant changes in individuals’ daily routines, namely: (i) interfering with their ability to work; (ii) compromising their family/social roles; and (iii) hindering their involvement in leisure activities [10]. Therefore, it is important to understand whether health care delivery provides "value" for the patients themselves [11], as well as to realize patients’ concerns, hopes and expectations, rather than reducing their deficiencies and disabilities adopting only a biological and functional perspective [4, 12]. This approach has motivated researchers and organizations worldwide to understand how comorbidities truly affect individuals' life, mainly in older age groups, and how these individuals self-report their disease burden [13].

Based on the above, an assessment that is capable of capturing the dynamic interaction between the individuals' external living conditions and their internal perception of these conditions has been progressively claimed [14, 15]. This is one of the main reasons why measuring individuals' quality of life (QOL) is becoming increasingly popular, with the aim of capturing individuals’ perception of their own health and their hopes, expectations and feelings after the delivery of health care [12, 16]. Additionally, QOL assessment has also been considered an important outcome of healthcare delivering in different clinical scenarios [17]. Therefore, identifying why individuals have poor QOL may help to ensure adequate health personnel for treatment and rehabilitation interventions, with the aim of improving individuals’ physical and mental health, level of independence, social relations, personal convictions and beliefs. On the other hand, QOL assessment may favor the implementation of well-informed public health policies, which may contribute to the avoidance of acute health scenarios in terms of older individuals’ diseases and disabilities [15]. In short, QOL assessment may lead to the promotion of an healthy life and populations’ well-being, especially among older individuals, who represent the population group that reports the greatest burden of noncommunicable diseases [7, 16].

The strong worldwide interest in measuring QOL led the World Health Organization (WHO), through the World Health Organization Quality of Life (WHOQOL) group, to develop the most comprehensive concept of QOL found in the scientific literature, favoring a transcultural, multidimensional and a subjective view of this construct [18]. To enable a QOL measurement, two instruments of international scope were developed by the WHOQOL group [19] in collaboration with several institutional groups worldwide: (i) the WHOQOL-100, which comprises 24 facets on QOL (four items per each facet) and one general facet (four items) [20] and (ii) the WHOQOL-BREF, which comprises the same 24 facets as in the former instrument, but only one item per each facet, and one general facet – GF (two items) [21]. The WHOQOL-BREF is considered the abbreviated version of the first instrument [22, 23].

A group of Portuguese (European) researchers, in collaboration with the WHOQOL group, accomplished the translation and validation of both international versions of the instruments in the Portuguese language between early 2004 and 2006, to allow others to research the QOL of Portuguese citizens using the WHOQOL instruments [19]. Similar translation/validation work was also performed in many cultures and languages [22, 23]. As a result of the important work conducted by the aforementioned group of Portuguese researchers, the following Portuguese (PT) versions of both international instruments were published: the WHOQOL-100(PT) [24] and the WHOQOL-BREF(PT) [25]. These versions have the same factorial models as the corresponding international versions, with the latter being the instrument under discussion in this article. During the translation/validation of the WHOQOL-BREF international instrument in the Portuguese language, the aforementioned group of Portuguese researchers collected data from two samples from a younger-adult population, in accordance with the standards issued by the WHOQOL group for the sample composition: (i) the “healthy group” (the average age was 40 years old) and (ii) the “clinical group” (comprising 50% of individuals aged equal to or greater than 45 years old), with this latter group composed of individuals who had health conditions and who were registered in three public health units of Coimbra city in different medical specialties [26].

However, reliability and validity are not fixed properties of a scale [27]. For example, as stated by Keszei et al. [28, page 321], “It is wrong to talk about the reliability of a scale, as opposed to the reliability of a scale used with a specific population for a given purpose.”, and “A scale that is reliable in one set of circumstances may not be reliable under different conditions.”. Based on this, the psychometric properties of the WHOQOL-BREF(PT) were re-evaluated in a sample of elderly citizens (individuals with higher burden of noncommunicable diseases, mostly unemployed and retired) residing in a rural area (substantial proportion of illiterates, living in a region with a scarce transportation network that difficult the access to healthcare services), rather than in a sample of a younger-adult population living in a major city, to assess the psychometric soundness of the instrument [29]. Moreover, a comparison of the psychometric properties of the WHOQOL-BREF(PT) obtained in this article to those attained by the aforementioned group of Portuguese researchers [26] and those reported in Skevington et al. [23] was also undertaken, to ascertain how different or similar these properties might be.

Since QOL is a multidimensional construct, comprising several dimensions of individuals’ life, some authors have found that it may be important to summarize this construct, instead of reporting the findings of QOL domains separately, as it may allow for a cohesive picture of QOL [30]. As this topic was not considered during the translation/validation of the WHOQOL-BREF international version in the Portuguese language, or even by the WHOQOL group, a strategy to determine an overall QOL scoring, here designated as QOL_24_ and comprising the 24 facets on QOL, is proposed in this article as novelty.

In short, the novel contributions of this article, specifically those related to the work previously undertaken by the group of Portuguese researchers on QOL [26], are listed as follows: (i) to re-evaluate the psychometric properties of the WHOQOL-BREF(PT) in a sample of elderly citizens, namely: the goodness of fit, scale’s internal consistency, convergent and discriminant validity, through a first-order confirmatory factor analysis (first-order CFA); (ii) to determine the QOL_24_ scoring, through a hierarchical second-order confirmatory factor analysis (H-second-order CFA); and (iii) to infer about the correlation level between the QOL_24_ and GF latent variables (latent factors), through a more generic structural equation modeling (SEM). All the research work was carried out using a sample of elderly citizens (more than or equal to 65 years old) residing in the community, in their own homes or at family members’ or friends’ homes.

## Methods

### Ethical considerations

The Health Ethics Committee of the Local Health Unit of Baixo Alentejo (HECLHUBA) [31] approved the study protocol on July 6th, 2014. The decision was published in the minutes of meeting issued by the HECLHUBA board of directors, with the reference number 2/2014. The HECLHUBA also approved the study design, methods, interview procedures and the informed consent form to be presented to each participant. Moreover, all the research methods were performed in full accordance with the statements included in the operating regulations of HECLHUBA [32], a document that was developed in accordance with the Helsinki Declaration with the aim of protecting the dignity, privacy and freedom of the participants [33].

### Study area

The Baixo Alentejo (BA) region [34] was chosen for this research because: (i) it presents a complex, worrisome and heterogeneous sociodemographic situation in regard to population aging; (ii) it has a significant rural context since it is located in the south-central area of mainland Portugal; (iii) it has a very low population density, with geographic distances between villages ranging from 25 to 120 km; and (iv) it has a limited and insufficient public transportation network, causing serious difficulties in terms of the displacement of elderly citizens living on their own means.

### Sample size

This research involved older individuals (more than or equal to 65 years old) who were residing in their own homes or at family members’ or friends’ homes. All the participants were registered in the database of the Local Health Unit of Baixo Alentejo (LHUBA), comprising 32,893 elderly citizens [35].

To determine the appropriate sample size *n* and the stratum sample sizes *n*_*1*_ to *n*_*6*_ (by gender – male and female – and by age group – 65 to 74, 75 to 84, and 85 years or older), the formulae proposed by Scheaffer et al. [36, page 128] were adopted. More precisely, the equation (5.10) was used to determine the sample size *n*, while the equation (5.9)—called the Neyman allocation—was employed to obtain *n*_*1*_ to *n*_*6*_. Additionally, the stratum standard deviations were calculated as $\sqrt {{\varvec{\pi}}_{{\varvec{i}}} \times \left( {1 - {\varvec{\pi}}_{{\varvec{i}}} } \right)}$, see Oliveira [37 pp. 65–66], where π_*i*_ denotes the population proportion of the *i*th stratum, set as 0.5 in this article since it represents the worst scenario in terms of *n*. Moreover, the sample size *n* was determined with a bound on the error of estimation equal to 4.5% (empirically chosen). Finally, the calculated sample size was 468 individuals. However, the final sample totaled 351 older individuals (163 males and 188 females who were randomly selected from the entire LHUBA database) who signed the informed consent form and answered the instrument fully and correctly (with no missing data).

According to the “*Calculator: a priori Sample Size for Structural Equation Models*” [38, 39], for an anticipated effect size of 0.3 (a medium effect, based on Cohen [40]), a desired statistical power level of 0.8 (a reasonable value as stated by Westland [41]), 4 latent variables (latent factors) and 24 instrument items (the 2 items belonging to the GF were not included in this calculation because they did not belong to the first-order and H-second-order CFA models), as well as a *p-value* of 5% [41], the a priori minimum sample size is 200 (even if a desired statistical power level of 0.9 is adopted, the resulting calculation remains unchanged).

The age of the respondents ranged from 65 to 101 years old. The average age and standard deviation were of 78.1 and 7.86, respectively.

### Inclusion criteria

The adopted inclusion criteria were as follows: individuals who (i) were aged 65 or older; (ii) were interested in participating in the study; (iii) were residing in BA region in their own homes or at family members’ or friends’ homes; and (iv) were able to make their own decisions if they were sick or were hospitalized due to acute, short-term health care needs.

### Data collection

Data were collected between January 2016 and April 2017 at the elderly citizens’ homes or their family members’ or friends’ homes by teams of health professionals from LHUBA. A structured interview methodology was adopted using the WHOQOL-BREF(PT) instrument [25]. All health professionals from LHUBA involved in the study received prior training on how to conduct the interviews, how to provide all necessary clarifications regarding the content of the instrument used, and how to avoid missing data. Before each interview, each health professional provided an informed consent form to the respondent or his/her family. Information on the study objectives was provided in full to the respondents and/or their families, and they were informed of the confidentiality and anonymity of the data.

### Instrument

The WHOQOL-BREF(PT) instrument comprises 24 facets (one item per each facet) clustered into four domains [25]: (i) Physical Health (7 facets), (ii) Psychological (6 facets), (iii) Social Relationships (3 facets), and (iv) Environment (8 facets). Additionally, it also includes a GF, which comprises 2 items of a general nature: (i) first, an item asking how the respondent would rate his/her quality of life (labeled as “overall QOL” in Skevington et al. [23] and G1 in this article), and (ii) second, an item asking how the respondent would rate his/her satisfaction with his/her health (labeled as “overall health” in Skevington et al. [23] and G4 in this article). All WHOQOL-BREF(PT) facets are measured using a five-point Likert scale (1 to 5), with the F1.4, F11.3 and F8.1 measured on an inverted scale. A list of all the facets is included in the appendix. The main characteristics of the WHOQOL-BREF(PT) instrument are as follows [21]: (i) it is cross-cultural; (ii) it can be applied to individuals living in different contexts; (iii) it is capable of capturing individuals’ own views of their well-being; and (iv) it should be self-administered if participants reveal enough reading skills.

### Statistical procedures

The goodness of fit, scale’s internal consistency, convergent and discriminant validity were assessed by employing a first-order CFA model (neither developed by Canavarro et al. [26] nor by Skevington et al. [23]) rather than the calculation of Pearson’s correlation coefficients between QOL domains, using the *lavaan* package of version 0.6–6 [42] for R statistics software of version 4.0.2 [43]. The diagonally weighted least squares (DWLS), specifically designed when neither the normality assumption nor the continuity property is considered plausible, was the estimator of the model parameters employed in the *lavaan* package for ordinal data [44], in which the diagonal weight matrix is used instead of the full weight matrix [45].

The goodness of fit was evaluated using the following fit indexes: (i) comparative fit index (CFI); (ii) Tucker-Lewis index (TLI); (iii) root mean square error of approximation (RMSEA); (iv) 90 percent confidence interval for the population RMSEA (RMSEA_CI(90%)_); and (v) test of the null hypothesis that the population RMSEA would be no greater than 0.05, often referred as PClose. The scale’s internal consistency was evaluated with Cronbach’s alpha coefficients (usually $\alpha_{c}^{j} >$ $0.6$ for the *j*th latent factor) and an alternative measure, i.e., the composite reliability (CR), calculated using the standardized factor loadings (λ_ij_) of the *i*th reflective items of *j*th latent factor (usually CR_j_ ≥ 0.7, as recommended by Marôco [46, page 183]). Convergent validity was evaluated by the average variance extracted (AVE) of each latent factor (usually AVE_j_ ≥ 0.5, again as recommended by Marôco [^46^, page 184]). Finally, the discriminant validity was assessed based on the positive validity of the expression $\left[ {\left( {\sqrt {AVE_{l} } > \rho_{lk} } \right) \wedge \left( {\sqrt {AVE_{k} } > \rho_{lk} } \right)} \right]$, where *lk* represents the *l*th and *k*th latent factors and $\rho_{lk}$ is their standardized correlation coefficient [46].

A H-second-order CFA was implemented as in Skevington et al. [23] (not developed by Canavarro et al. [26]), to determine the score of QOL_24_ latent factor. The correlation level between QOL_24_ and GF latent variables was assessed trough a more generic SEM (designated in this article as ${\text{SEM}}_{{{\text{QOL}}_{24} - {\text{GF}}}}$) rather than a multilinear regression analysis (with the QOL domains as the endogenous variables and GF as the exogenous variable) as in Canavarro et al. [26]. The goodness of fit of both the H-second-order CFA and ${\text{SEM}}_{{{\text{QOL}}_{24} - {\text{GF}}}}$ models was evaluated using the same fit indexes previously mentioned for the first-order CFA. The scoring of the four QOL domains and QOL_24_ was performed based on the factor score weights (*fsw*_*i*_) – one per item – predicted from the H-second-order CFA model, resulting in weighted averages, where the *fsw*_*i*_ values were used as weights after their standardization to 100%.

Nonparametric tests of the means (paired samples) were also conducted to compare the sample scores for the QOL domains calculated through the two different strategies: (i) unweighted average, as in Canavarro et al. [26] and Skevington et al. [23], and (ii) weighted averages, as proposed in this article as novelty.

## Results

### Sample size allocation

Table 1 lists the results of the sample size allocation procedure for the “calculated” sample size and the real number of “participants”. Following Westland [41], who stated that “*for non-normal data such as Likert scale data, sample sizes of at least one to two magnitudes larger may be needed*”, the number of 351 participants involved in this research work was in accordance with the author's statement because it was approximately 175.5% more than 200 (see the penultimate paragraph of *Sample size* subsection).

**Table 1:** Results of the sample size allocation procedure

Variables	Calculated		Participants	
	n	%	n	%
*Male*	195	41.7	163	46.4
65–74	93	47.7	64	39.3
75–84	77	39.5	58	35.6
85 or more	25	12.8	41	25.1
*Female*	273	58.3	188	53.6
65–74	111	40.7	68	36.2
75–84	111	40.7	77	40.9
85 or more	51	18.6	43	22.9

### First-order CFA

**Fig. 1:**
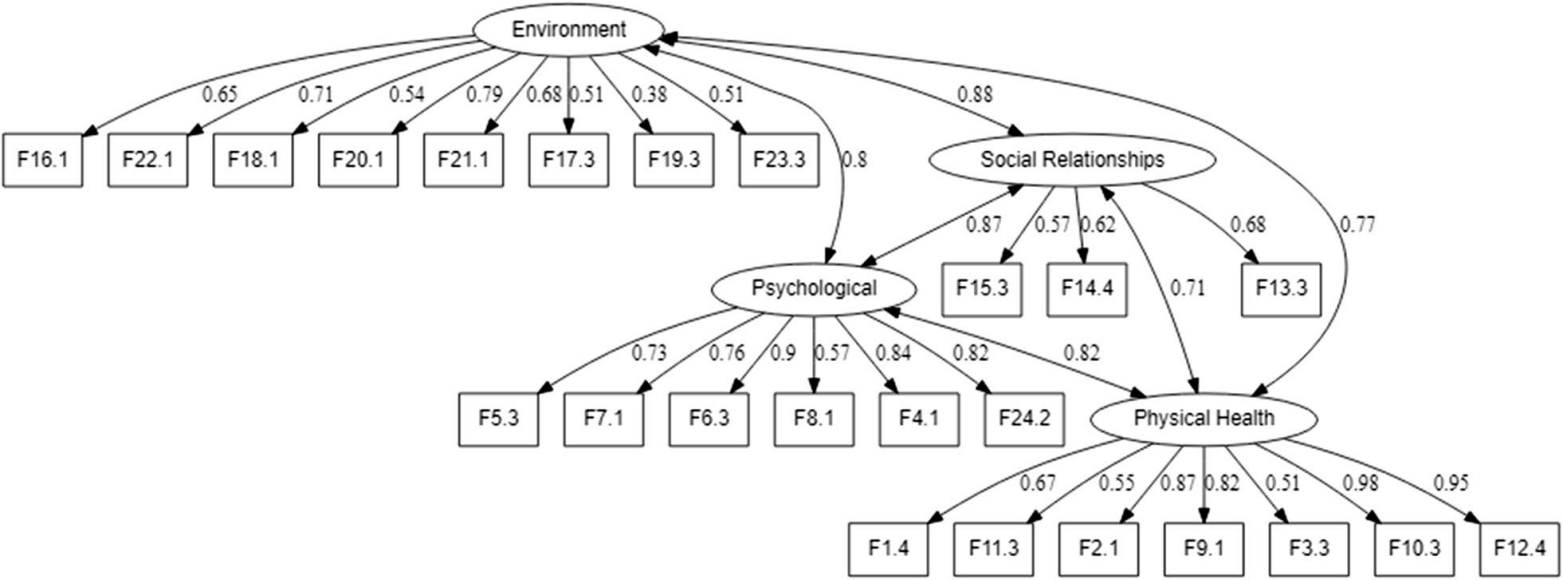
First-order CFA model of the WHOQOL-BREF(PT)

The overall fit of the first-order CFA model (see the model in Fig. 1) was classified as “almost very good” (see Table 2). The standardized factor loadings (λ_i_) were 0.38 ≤ λ_i_ ≤ 0.98 (see Fig. 1, all were statistically highly significant, *p* < 0.001), with an average of 0.69, and only one λ_i_ below the threshold value of 0.5 (λ_F19.3_ = 0.38). For the assessment of the scale’s internal consistency (see Table 3) based on the $\alpha_{c}^{j}$ measure (see Table 3), all domains presented values above the admissible threshold of 0.6. However, considering the reliability analysis based on the CR measure, only the Social Relationships domain presented a value lower than 0.7 (this index was not available neither in Canavarro et al. [26] nor in Skevington et al. [23]). With respect to the convergent validity (see Table 3), the Physical Health and Psychological domains presented values above the recommended threshold of 0.5, although values of 0.3 ≤ AVE_j_ < 0.5 may be considered “acceptable” in the case of exploratory research [46, p. 184] (this index was not available neither in Canavarro et al. [26] nor in Skevington et al. [23]). The first-order CFA model presented no discriminant validity since the results of all possible expressions $\left[ {\left( {\sqrt {AVE_{l} } > \rho_{lk} } \right) \wedge \left( {\sqrt {AVE_{k} } > \rho_{lk} } \right)} \right]$ were “false”. Regarding the standardized correlation coefficients between the QOL domains (*ρ*_*lk*_), all were statistically highly significant (*p* < 0.001, see Table 3).

**Table 2:** Results of the robust solution regarding the three SEM models

Indexes	Index values			Qualitative classification^a^
	First-order CFA	H-second-order CFA	${\text{SEM}}_{{{\text{QOL}}_{24} - {\text{GF}}}}$	
χ^2^	1022.622	1042.076	1131.384	–
df	246	248	294	–
*p value* (χ^2^)	< 0.001	< 0.001	< 0.001	–
CFI	0.949	0.948	0.949	Almost very good
TLI	0.943	0.942	0.943	Almost very good
RMSEA	0.095	0.096	0.090	Admissible
RMSEA_CI(90%)_	[0.089; 0.101]	[0.090; 0.102]	[0.085; 0.096]	–
PClose	< 0.001	< 0.001	< 0.001	–

^a^According to Marôco [46]

**Table 3:** Measures of internal consistency (CR and $\alpha_{c}^{j}$), AVE_j_ and correlation coefficients between the QOL domains (values reported in Canavarro et al. [26] are shown in square brackets, while the ones reported in Skevington et al. [23] are shown in round brackets, if available)

	Physical health	Psychological	Social relationships	Environment
$\alpha_{c}^{j}$	0.90 [0.87] (0.82)	0.89 [0.84] (0.81)	0.64 [0.64] (0.68)	0.81 [0.78] (0.80)
CR_j_	0.88	0.86	0.59	0.78
AVE_j_	0.614	0.606	0.390	0.374
Physical health	1.00	–	–	–
Psychological	0.82** [0.55**]^a^	1.00	–	–
Social relationships	0.71** [0.56**]^a^	0.87** [0.72**]^a^	1.00	–
Environment	0.77** [0.57**]^a^	0.80**	0.88**	1.00

**p < 0.001; ^a^Pearson’s correlation coefficients as published in Canavarro et al. [26]

### H-second-order CFA

**Fig. 2:**
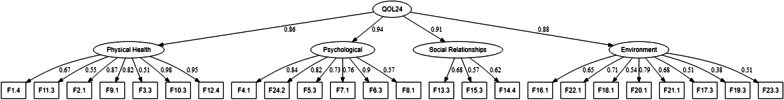
H-second-order CFA model of the WHOQOL-BREF(PT)

A H-second-order CFA model was developed as in Skevington et al. [23], by positioning a higher-order factor, called QOL_24_, as shown in Fig. 2. As suggested by the values in Table 2, the H-second-order CFA also showed an “almost very good” fit, with the QOL_24_ latent factor operationalized by four latent variables (Physical Health, Psychological, Social Relationships and Environment), in which each of the four latent variables was operationalized by several observed variables (comprising the 24 facets of the instrument). Regarding the standardized factor loadings (λ_i_), the ones reported in Skevington et al. [23] (shown in round brackets) were somewhat similar to those obtained within this article, i.e., 0.38(0.40) ≤ λ_i_ ≤ 0.98(0.82), see Fig. 2. With respect to the correlations between QOL_24_ and the four domains (designated in this article as *ρ*_*j*_), all were strong and statistically highly significant (*p* < 0.001), and slightly higher than those reported in Skevington et al. [23] (shown in round brackets): (i) *ρ*_Physical Health_ = 0.86(0.87); (ii) *ρ*_Psychological_ = 0.94(0.95); (iii) *ρ*_Social Relationships_ = 0.91(0.83) and (iv) *ρ*_Environment_ = 0.88(0.84), see Fig. 2. The ${\text{CR}}_{{{\text{QOL}}_{24} }}$ and ${\upalpha }_{{\text{c}}}^{{{\text{QOL}}_{24} }}$ measures showed very good values, respectively 0.943 and 0.940 (${\upalpha }_{{\text{c}}}^{{QOL_{24} }}$ = 0.92, reported only in Canavarro et al. [26]). Finally, ${\text{AVE}}_{{{\text{QOL}}_{24} }} = 0.807$, which was above the admissible threshold of 0.5 (not reported neither in Canavarro et al. [26] nor in Skevington et al. [23]).

$$ {\text{SEM}}_{{{\text{QOL}}_{24} - {\text{GHF}}}} $$

**Fig. 3:**
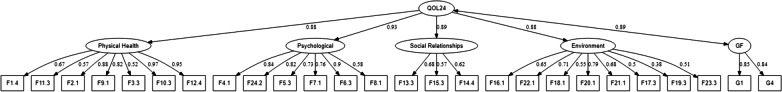
${\text{SEM}}_{{{\text{QOL}}_{24} - {\text{GF}}}}$ model of the WHOQOL-BREF(PT)

Regarding the standardized factor loadings (λ_i_), the values were 0.38 ≤ λ_i_ ≤ 0.98 (all were statistically highly significant, *p* < 0.001), with an average of 0.69, and only one λ_i_ was below the threshold value of 0.5 (λ_F19.3_ = 0.38). As described in the “Methods” section, the WHOQOL-BREF(PT) includes a GF comprising two items (G1 and G4). A third SEM model (see Fig. 3) was developed to investigate the correlational level between the QOL_24_ and GF latent factors. The fit indexes for this model were very similar to those of the previous two CFA models, as listed in Table 2, also classified qualitatively as “almost very good”. The correlation level between QOL_24_ and GF was strong (*ρ* = 0.89; *p* < 0.001).

### Descriptive statistics

**Fig. 4:**
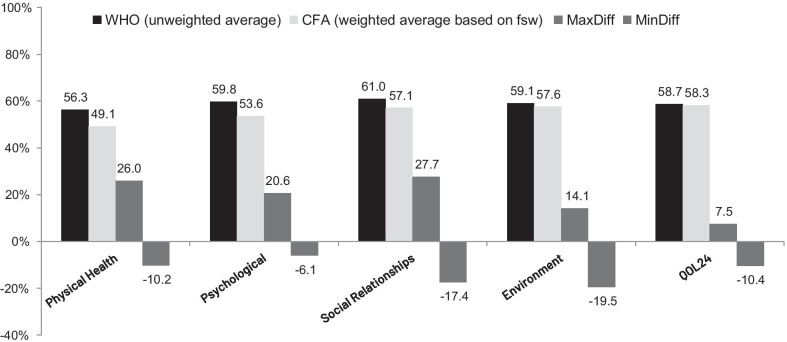
Comparison between the average scores of the entire sample based on WHO strategy (unweighted average) and using the *fsw*_*i*_ predicted from the H-second-order CFA model (weighted average)

Table 4 summarizes the descriptive statistics regarding the four QOL domains, QOL_24_ and GF, while Fig. 4 shows the results of the average scoring obtained from two different strategies: unweighted average (black bars) and the use of the *fsw*_*i*_ (weights in the weight average strategy) predicted from the H-second-order CFA model (bars depicted in light gray). The results of nonparametric tests (paired data) seem to suggest that the differences found in the scores of each of the four QOL domains and the QOL_24_ (between the unweighted and weighted averages) were not due to chance, because each difference was statiscally highly significant (*p* < 0.001). However, no evidence was found to support the rejection of the null hypothesis in relation to the GF factor regarding this topic.

**Table 4:** Descriptive statistics of the latent factors of the WHOQOL-BREF(PT)

Domains	Mean	Median	SD	Skewness	SE_Sk_	Kurtosis	SE_Ku_	Min	Max
Physical health	3.254	3	1.042	− 0.118	0.049	− 0.823	0.099	1	5
Psychological	3.392	4	0.901	− 0.367	0.053	− 0.296	0.107	1	5
Social relationships	3.439	4	0.842	− 0.444	0.075	0.184	0.151	1	5
Environment	3.365	4	0.929	− 0.538	0.046	− 0.188	0.092	1	5
QOL_24_	3.348	3	0.948	− 0.361	0.027	− 0.412	0.053	1	5
GF	3.056	3	0.927	− 0.154	0.092	− 0.607	0.184	1	5

## Discussion

The first objective proposed in this article was to assess the goodness of fit, internal consistency, convergent and discriminant validity of the WHOQOL-BREF(PT) based on a first-order CFA model (see Fig. 1), which is considered an appropriate strategy if a factorial model is already available [47]. The findings reported in the previous section seem to suggest that the WHOQOL-BREF(PT) instrument showed an “almost very good” goodness of fit. All the standardized factor loadings were above the threshold of 0.3, as recommended by Fleck [14]. An adequate internal consistency (similarly reported in Canavarro et al. [26] and Skevington et al. [23]) was also reached. Regarding the lower value of ${\upalpha }_{{\text{c}}}^{{\text{Social Relationships}}}$, researchers usually explain such a finding to be a result of the small number of items included within this QOL domain [14, 18, 26], which is in line with the fact that this metric is affected by the number of items in a latent factor [23, 46]. The reported results also seem to suggest that a tolerable convergent validity was achieved. Strong correlations between first-order factors (higher than those reported in Canavarro et al. [26], see Table 3) and lower AVE values were obtained. Since the AVE_j_ values were less than the square of the correlation between the latent factors involved, the instrument did not show discriminant validity. While the discriminant validity based on CFA follows the Fornell–Larcker criterion [47], Canavarro et al. [26] adopted the strategy proposed by Nunnally and Bernstein [46]. However, because the Fornell–Larcker criterion is based on the correlations between QOL domains, it can help infer the extent to which the domains are related to each other [30]; i.e., (i) strong correlations among domains may suggest that changes in a domain can have significant impacts on other domains, while (ii) weak correlation among domains may suggest that changes in one domain can have little or no impact on other domains. Considering this inference and the strong correlations between first-order latent factors, it is possible to establish a relationship between the findings obtained within the scope of this article and the results reported by Van Leeuwen et al. [15, page 34], who stated: “*However, it is important that service providers and care professionals realize that the QoL domains are strongly intertwined, meaning that changes in one domain likely affect other QoL domains.*”. An additional comment is made regarding the discriminant validity achieved by Canavarro et al. [26] and Skevington et al. [23]. The authors evaluated a successful discriminant validity in their research work, such as the instrument's ability to differentiate individuals belonging to the “healthy” or “clinical” (patients) groups, based on the results of a Student's *t*-test used to compare the average scores of the QOL domains between the two groups. Therefore, it is difficult to compare the two results, those reached by Canavarro et al. [26] and Skevington et al. [23] and the ones achieved within the scope of this research work, since they were obtained based on different statistical methodologies.

The H-second-order CFA (see Fig. 2), which was more related to the research carried out by Skevington et al. [23] (not reported in Canavarro et al. [26]) and aimed to predict the QOL_24_ scoring, was also undertaken. The results of fit indexes seem to suggest that the proposed model adequately fit the sample data of elderly citizens residing in a rural area, suggesting that WHOQOL-BREF(PT) may be considered a psychometrically sound instrument to assess the QOL of this population group [29]. Since the results in Skevington et al. [23] were presented in terms of four different samples (two split-half samples of the data, as well as the “sick persons” and “well persons” sub-samples), it is difficult to compare the fit indexes achieved by the authors with the ones obtained within the scope of this research work. Regarding the correlations between the QOL_24_ latent variable and each domain, the correlations presented in the *H-second-order* subsection of this article were slightly higher than those reported in Skevington et al. [23], especially for the Social Relationships domain, again suggesting that QOL among older respondents is a construct that strongly relates several dimensions of their life, even more than in other population group [16]. Adopting the Aristotle’s statement: “*the whole is not the same as the sum of its parts*”, it may be interesting to obtain the QOL_24_ scoring based on the entire set of QOL domains because they are strongly intertwined and may allow for a cohesive picture of this construct [30]. Therefore, a scoring strategy for the four domains and QOL_24_ is presented in this article as novelty by determining the scores as weighted averages and using the *fsw*_*i*_ values as weights. With respect to the results of nonparametric tests (paired samples), used to compare the scoring strategies by unweighted and weighted averages, they seem to suggest that all the differences observed between the two scoring strategies of the four domains and QOL_24_ (see Fig. 4 for details) were not due to chance. Additionally, in the analysis of individual scores (for each respondent separately), differences were also observed (see the small positive and negative bars depicted in dark gray in Fig. 4). Although the differences between the two scoring strategies may be easily detected through the employment of nonparametric tests (paired samples), their evaluation in terms of how they really corresponded to the individuals’ QOL assessments may not seem to be an easy task. In fact, deciding between the two types of scoring (e.g., 59% based on the unweighted average or 48% based on the weighted average through the use of *fsw*_*i*_ values), i.e., deciding which is the strategy that truly corresponds to the individual’s QOL_24_ scoring, probably is a task that can only be taken by a group of experts (e.g., including health professionals, people from the community, healthy individuals and individuals presenting comorbidities). This methodology could allow expert consensus to be reached about which is the best matched scoring: the scoring based on an unweighted average strategy or the scoring proposed in this research work as novelty. Although the use of an unweighted average can be advantageous since it results in an invariant scale’s scoring [42, 47], the use of *fsw*_*i*_ values can allow for certain peculiarities of the QOL domains, which were captured by the structural equation modeling, be reflected in the scoring [46].

The correlation between the QOL_24_ and GF latent variables, which was not reported in Skevington et al. [23], was investigated in this article using a more generic SEM (see Fig. 3 for details). However, this was a different strategy than the one reported in Canavarro et al. [25], where a multilinear regression analysis was undertaken by the authors (with the QOL domains as the endogenous variables and GF as the exogenous variable) to identify which domains were considered the best predictors of the GF. In their research work, the Physical Health domain was considered the best predictor of the GF, with a variance explained of 52.2%, followed by the Psychological, Environment and Social Relationships domains, in descending order of the variance explained. Concerning the ${\text{SEM}}_{{{\text{QOL}}_{24} - {\text{GF}}}}$ model undertaken in this article, the correlation between the QOL_24_ and GF latent variables was found to be quite strong, suggesting that the GF, which comprises the G1 and G4 items, may be a latent factor that can be used as a generalized measure of QOL. This finding indicates that concurrent validity between these two latent variables was reached, thus accomplishing the third objective of this research work. In addition to this finding, it is important to mention that the use of the GF factor (only 2 items) instead of the full set of 24 items may be considered, although with some caution (only if concurrent validity between QOL_24_ and GF latent factors is reached), especially in large clinical settings and epidemiological surveys, where the respondent burden must be minimized, or in cases where it is necessary to reduce the number of variables in a model [22, 23]. However, more studies must be undertaken to check the validity of using the GF as a generalized measure of QOL_24_.

## Conclusions

This article presents a new perspective regarding the assessment of the WHOQOL-BREF(PT) psychometric properties based on SEM models and applying the instrument to elderly citizens residing in a rural area with a very low population density in mainland Portugal (Europe). The findings in this research seems to suggest that the WHOQOL-BREF(PT) is a psychometrically sound instrument that can be used to assess the QOL of the considered population sample, although discriminating validity could be a psychometric property that may be difficult to be reached. Summarizing the domain measures into the QOL_24_ scoring may give a cohesive picture of QOL, especially if strong correlations among domains are found, which was the case in this article, corroborating the finding that QOL is a construct that strongly involves several dimensions of older individuals’ life. Concurrent validity between QOL_24_ and GF was also achieved, which may suggest that the GF can be used as a generalized QOL measure, although with some caution.

### Limitations and future research

The number of respondents, which was lower than expected, was one of the weaknesses of this study, leading to a higher sample error than expected. Another limitation was related to the fact that this was not a longitudinal study, so long-term follow-up was not carried out; long-term follow-up is an important strategy that allows the examination of the instrument’s sensitivity to capture changes in health states over time. Moreover, it was not possible to perform test–retest reliability and longitudinal time invariance tests. Since there are no reference values for “good” or “bad” QOL, it is therefore not possible to carry out a qualitative assessment of this construct. Given the usefulness of measuring this construct in clinical assessment, further research is recommended to create a normative basis to promote its construction. Furthermore, the weighted scoring of QOL_24_ should be validated by a group of experts (e.g., including health professionals, people from the community, healthy individuals and individuals presenting comorbidities) to reach expert consensus about this topic.

## Data Availability

All data and materials in this article can be obtained by contacting the corresponding author, Henrique Oliveira (hjmo@lx.it.pt).

## Appendix

### List of WHOQOL-BREF(PT) facets/items

General facet:

G1. Overall quality of life

G4. Overall health

Physical Health domain:

F1.4 Pain and discomfort

F2.1 Energy and fatigue

F3.3 Sleep and rest

F9.1 Mobility

F10.3 Activities of daily living

F11.3 Dependence on medication or health care

F12.4 Work capacity

Psychological domain:

F4.1 Positive feelings

F5.3 Thinking, learning, memory and concentration

F6.3 Self-esteem

F7.1 Body image and appearance

F8.1 Negative feelings

F24.2 Spirituality/religion and personal beliefs

Social relationships domain:

F13.3 Personal relations

F14.4 Practical social support

F15.3 Sex

Environment domain:

F16.1 Physical safety and security

F17.3 Home environment

F18.1 Financial resources

F19.3 Health and social care: availability and quality

F20.1 Opportunities to acquire new information and skills

F21.1 Recreation and leisure

F22.1 Physical environment (pollution/noise/traffic/climate)

F23.3 Transport

## Abbreviations

AVE: Average variance extracted
BA: Baixo Alentejo (the study region)
BD: Burden of disease
CFA: Confirmatory factor analysis
CFI: Comparative fit index
CHRC: Comprehensive Health Research Centre
CR: Composite reliability
DWLS: Diagonally weighted least squares
*fsw*: Factor score weight
GF: General facet
HECLHUBA: Health Ethics Committee of Local Health Unit of Baixo Alentejo
LHUBA: Local Health Unit of Baixo Alentejo
MI: Modification index
PClose: Test of the null hypothesis that the population RMSEA would be no greater than 0.05
QOL: Quality of life
RMSEA: Root mean square error of approximation
RMSEA_CI(90%)_: 90 Percent confidence interval for the population RMSEA
SE_Sk_: Standard error of skewness
SE_Ku_: Standard error of kurtosis
SEM: Structural equation model
SD: Standard deviation
TLI: Tucker-Lewis index
WHO: World Health Organization
WHOQOL: World Health Organization Quality of Life
WHOQOL-100: The international version of the instrument issued by the World Health Organization (in the English language)
WHOQOL-100(PT): Version of the WHOQOL-100 instrument in the Portuguese language
WHOQOL-BREF: The international version of the instrument issued by the World Health Organization (in the English language)
WHOQOL-BREF(PT): Version of the WHOQOL-BREF instrument in the Portuguese language
